# Age-related neurogenesis decline in the subventricular zone is associated with specific cell cycle regulation changes in activated neural stem cells

**DOI:** 10.1038/srep21505

**Published:** 2016-02-19

**Authors:** Mathieu Daynac, Lise Morizur, Alexandra Chicheportiche, Marc-André Mouthon, François D. Boussin

**Affiliations:** 1 CEA DSV iRCM SCSR, Laboratoire de Radiopathologie, UMR 967, F-92265 Fontenay-aux-Roses, France; 2 INSERM, UMR967, F-92265 Fontenay-aux-Roses, France; 3 Université Paris Diderot, Sorbonne Paris Cité, UMR 967, F-92265 Fontenay-aux-Roses, France; 4 Université Paris Sud, UMR 967, F-92265 Fontenay-aux-Roses, France

## Abstract

Although neural stem cells (NSCs) sustain continuous neurogenesis throughout the adult lifespan of mammals, they progressively exhibit proliferation defects that contribute to a sharp reduction in subventricular neurogenesis during aging. However, little is known regarding the early age-related events in neurogenic niches. Using a fluorescence-activated cell sorting technique that allows for the prospective purification of the main neurogenic populations from the subventricular zone (SVZ), we demonstrated an early decline in adult neurogenesis with a dramatic loss of progenitor cells in 4 month-old young adult mice. Whereas the activated and quiescent NSC pools remained stable up to 12 months, the proliferative status of activated NSCs was already altered by 6 months, with an overall extension of the cell cycle resulting from a specific lengthening of G_1_. Whole genome analysis of activated NSCs from 2- and 6-month-old mice further revealed distinct transcriptomic and molecular signatures, as well as a modulation of the TGFβ signalling pathway. Our microarray study constitutes a cogent identification of new molecular players and signalling pathways regulating adult neurogenesis and its early modifications.

Neurogenesis occurs throughout the adult lifespan in specific neurogenic zones of the mammalian brain, but mainly in the subventricular zone (SVZ) of the lateral ventricles and the subgranular zone (SGZ) of the hippocampus^1^,^2^. Adult neurogenesis within the SVZ is conferred by a stock of quiescent neural stem cells (qNSCs)^3^ that can enter the cell cycle and convert into their activated form, expressing the EGFR protein^4^,^5^,^6^,^7^,^8^. Activated NSCs (aNSCs) successively give rise to transit amplifying cells (TACs)^9^, immature neuroblasts (Im. Nbs) and migrating neuroblasts (Mig. Nbs) that differentiate into neurons once they have reached the olfactory bulbs^10^,^11^. Most studies agree on a progressive reduction in the number of proliferating progenitor cells in the SGZ and SVZ, which explains the dramatic drop in the number of neurons that are produced during aging^12^,^13^,^14^,^15^,^16^. Middle-aged (12 months) or elderly mice (24 months) have been intensively studied to understand the underlying mechanisms. Although the pool of NSCs remains stable until middle age^17^,^18^, NSCs progressively lose their proliferative capacities^18^,^19^,^20^ and enter quiescence^16^,^21^. On the other hand, a dramatic loss of progenitor cells is observed with aging^15^,^18^,^22^,^23^. We have previously shown that both pools of qNSCs and aNSCs are maintained until middle age, but aNSCs proliferation is affected by a lengthening of their G_1_ phase through a TGFβ-dependent mechanism, leading to a decrease in neurogenesis^18^,^24^.

Surprisingly, few studies have investigated early events in the neurogenic niches from young adults. Some studies have shown a significant decline in bromodeoxyuridine (BrdU) incorporation in progenitor cells by 6 months, associated with a decrease by half of the number of colonies (neurospheres) produced by SVZ progenitors *in vitro*^13^,^15^.

Novel fluorescent-activated cell sorting (FACS)-based methods allow for the identification and isolation of SVZ cells and a better understanding of the evolution of the neurogenic niche during aging^4^,^5^,^6^,^25^,^26^. The cell surface carbohydrate Lewis-X (LeX) has been shown as a potent and reliable marker to label and sort NSCs in the adult SVZ^5^,^27^,^28^,^29^. Herein, we used our previously published FACS technique^5^,^29^ to follow different cell populations in the SVZ in 2- to 12-month old mice. We observed that although the NSC pool stayed constant, there was an early decrease in the number of progenitor cells and neuroblasts in young adult mice. We demonstrated a specific decrease in aNSC proliferation with a lengthening of their G_1_ phase of the cell cycle as early as 6 months. Finally, a microarray analysis using whole-genome arrays showed, for the first time, a clear cellular evolution in aNSCs from 2- and 6-month-old mice with distinct transcriptional profiles.

## Materials and Methods

### Animals

Two, 4, 6, 9 and 12 month-old C57Bl/6 mice were used in this study. The animals were maintained with access to food and water *ad libitum* in a colony room kept at a constant temperature (19–22 °C) and humidity (40–50%) on a 12:12-hour light/dark cycle. For cell cycle analysis, we used mice transgenic for fluorescence ubiquitination cell cycle indicator (FUCCI) chromatin licensing and DNA replication factor 1 (Cdt1)-red (FUCCI-Red), (Gem)-green (FUCCI-green), or (Cdt1)-red/(Gem)-green^30^. Animal experiments were approved by Comité d’Ethique en Expérimentation Animale, Direction des Sciences du Vivant, CEA (ref 12–034). All experiments were performed in accordance with the European Communities Council Directive of 22th September 2010 (EC/2010/63).

### Preparation of SVZ cells and FACS

Lateral ventricle walls containing cells from the SVZ were dissected and dissociated as previously described^5^,^29^. For DNA content analysis, dissociated cells were incubated with the vital DNA marker Hoechst 33342 (Sigma)^5^,^31^. The antibodies to identify different cell populations were the CD24 phycoerythrin [PE]-conjugated (rat IgG2b; 1:50 BD Biosciences), CD24 phycoerythrin-cyanine7 [PC7]-conjugated (Rat IgG2b; 1:100 Life Technologies), CD15/LeX fluorescein isothiocyanate [FITC]-conjugated (clone MMA, mouse IgM; 1:50 BD Biosciences), mouse anti-human LeX-antibody (1:50 BD Biosciences) and Alexa647-conjugated EGF ligand (1:200 Life Technologies), which were incubated as reported^5^. To perform absolute cell counts, single cell suspensions were transferred to tubes containing a calibrated number of fluorescent beads (TruCount tubes, BD Biosciences). Prior to FACS sorting with FUCCI-Green mice, LeX-positive and LeX-negative fractions were separated using MACS LS separation columns (Miltenyi Biotec). Immediately prior to FACS, propidium iodide (PI) or Hoechst 33258 was added to a final concentration of 2 μg/mL to label the dead cells. Cells were analysed on an LSRII (BD Biosciences) and sorted on an INFLUX cell sorter (BD Biosciences) as reported^5^,^29^. Sorting gates were drawn according to fluorescence-minus-one (FMO)-controls. The data were analysed with FlowJo data analysis software (Tree Star, Ashland, OR, USA).

### *In vitro* assays

For neurosphere cultures, FACS-purified populations were plated at a density of 700 cells/well in 24-well tissue culture plates (TPP, Switzerland) for 7 days. Cells were grown in NeuroCult NSC basal medium supplemented with a proliferation supplement (STEMCELL Technologies), 2 μg/mL of heparin, 20 ng/mL of EGF and 10 ng/mL of FGF-2 (Sigma). For live cell imaging, freshly sorted cells from FUCCI mice were plated at a density of 1–3 × 10^3^ cells/well on poly-D-Lysine (Sigma)-coated 96-well glass bottom culture plates (MatTek Corp., Ashland, MA) in neurosphere medium. Brightfield and fluorescent images for FUCCI fluorescence were captured through a Plan Apo VC 20X DIC objective (Numerical Aperture: 0.75) on a Nikon A1R confocal laser scanning microscope system attached to an inverted ECLIPSE Ti (Nikon Corp., Tokyo, Japan) maintained at 37 °C in a 5% CO_2_ atmosphere. FUCCI Red fluorescence was excited at 561 nm and collected using a 595/50 nm filter set. Images were acquired and analysed as previously described^18^,^29^.

### Determination of BrdU incorporation

Mice were initially injected intraperitoneally with 100 mg BrdU/kg body mass then given BrdU in their drinking water (1 mg/mL BrdU, 1% glucose) for 14 consecutive days until sacrifice. Cells were then FACS sorted as described above and plated without mitogens on poly-D-lysine- and laminin-coated 8-well glass slides (Millicell) for 2 hours and then fixed in 2% paraformaldehyde. For BrdU detection, cells were permeabilized for 5 minutes at RT in 0.5% Triton X-100 PBS. Incubation in blocking solution (PBS, 0.05% Tween 20, 4% BSA) at 37 °C for 1 hour was followed by a 30-minute incubation at 37 °C with anti-BrdU at 1/150 (GE Healthcare) in DNase incubation buffer (0.5X PBS, 30 mM Tris-HCl pH 8, 0.3 mM MgCl2, 0.5 mM 2-mercaptoethanol, 0.5% BSA and 10 μg/mL DNase I). After several washes, cells were incubated with an Alexa fluor-conjugated donkey secondary antibody at 1:500 (Invitrogen).

### RNA isolation, amplification and hybridization to microarray

Sorted cells from 4 independent experiments were immediately lysed in RLT buffer (QIAGEN), and total RNA was isolated with the RNeasy Micro Kit (Qiagen) with DNase treatment according to the manufacturer’s instructions. cDNA was synthesized and amplified using the Ovation Pico WTA System (NuGEN) before being hybridized on Affymetrix MOE430 2.0 arrays (45,000 probe sets) according to the Affymetrix standard protocols at PartnerShip (Evry, France). The data were quality controlled with the Expression Console software (Affymetrix) following normalization with the MAS5 algorithm.

### Data normalization and statistical analyses

Microarray data were normalized with GC-robust multi-array analysis (GC-RMA) using log2 transformed expression levels in Genespring GX12. Differentially expressed probes were filtered by an average expression greater than 50 in at least 1 population, a differential expression of at least 2-fold and a Student’s t-test p-value < 0.05. A statistical overrepresentation analysis of GO biological processes (p < 0.05) was performed with PANTHER software (http://www.pantherdb.org) using binomial statistics with Bonferroni correction. For hierarchical clustering, expression values for all biological replicates were clustered using Pearson correlation (centred) and complete linkage. The expression levels among differentially expressed probes that map to the same gene were averaged in heat maps depicting gene expression changes between aNSCs from 2- and 6-month old mice.

### Statistical analyses

Non-parametric Mann-Whitney test was conducted using StatView5 software (SAS Institute Inc., Cary, NC). Significance was set at p < 0.05. The data are expressed as the mean ± SEM.

## Results

### Early loss of progenitors from the SVZ starts at 4 months of age

**Figure 1:**
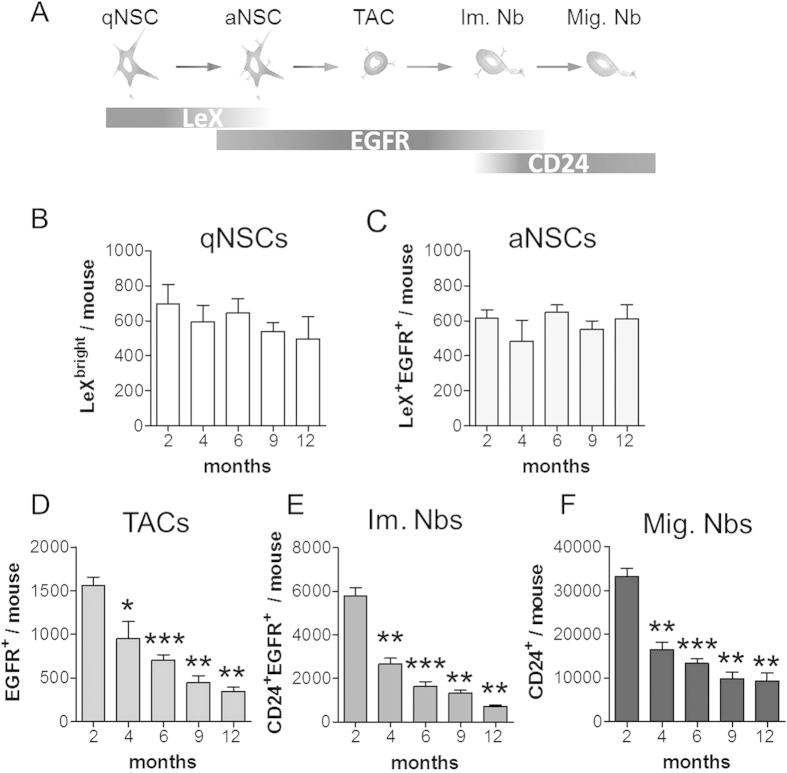
Decrease in the number of progenitor cells in the SVZ starts at 4 months. (**A**) LeX/EGFR/CD24 triple staining strategy allowing the purification of the main neurogenic populations from the adult SVZ as previously reported^5^. (**B–F**) Quantification of the 5 different SVZ populations of NSCs and their progeny by FACS using TrueCount microbead-calibrated tubes on mice aged 2 to 12 months. The data are represented as the mean ± SEM and were obtained from at least 4 independent mice for each time point. *p < 0.05, **p < 0.01, ***p < 0.001.

Although it is now well known that SVZ neurogenesis decreases in the aging adult mouse brain^13^,^16^,^18^,^23^, its early decline with age and the underlying molecular mechanisms have not been elucidated to date. To accurately determine the progression of SVZ neurogenesis with age, we quantified NSCs and their progeny by FACS at 2 (young adult), 4, 6, 9 and 12 months (middle-aged) based on the detection of 3 different cell surface markers, LeX, CD24 and EGFR, as previously reported^5^,^18^,^29^. These markers allow for the identification of the following 5 neurogenic cell populations (Fig. 1A; for clarity, the negative markers are not indicated): (i) LeX^bright^ cells, i.e., qNSCs; (ii) LeX^+^ EGFR^+^ cells, i.e., aNSCs; (iii) EGFR^+^ cells, i.e., TACs; (iv) EGFR^+^ CD24^+^, i.e., Im. Nbs; and (v) CD24^+^, i.e., Mig. Nbs. As shown in Fig. 1B,C, both the pools of qNSCs and aNSCs remained stable with age in the mouse SVZ, consistent with previous reports^16^,^18^,^21^. In contrast, the cell fractions containing their progeny decreased constantly from 2 to 12 months. Surprisingly, the absolute numbers of TACs, Im. Nbs and Mig. Nbs were already decreased at 4 months compared with 2 months by 39.0%, 53.8% and 50.5%, respectively (Fig. 1D–F). To evaluate the overall changes that occur within the SVZ niche, we quantified the proportion of each cellular population relative to the total number of cells in the SVZ (Fig. S1). Despite the sharp decrease in size of the SVZ at 4 months (Fig. S1A), the niche organization was relatively preserved except for a small decrease in the proportion of Im.Nbs (Fig. S1E). In contrast, the niche was dramatically altered after 6 months of age with a progressive over-representation of stem cells compared to progenitors and neuroblasts (Fig. S1B–F).

Together, these data show that the decrease in SVZ neurogenesis is a continuous process that starts very early during the adult mouse life, which progressively reduces the production of TACs and Nbs, whereas the NSC pool remains stable.

### The activated NSC cell cycle is altered at 6 months

**Figure 2:**
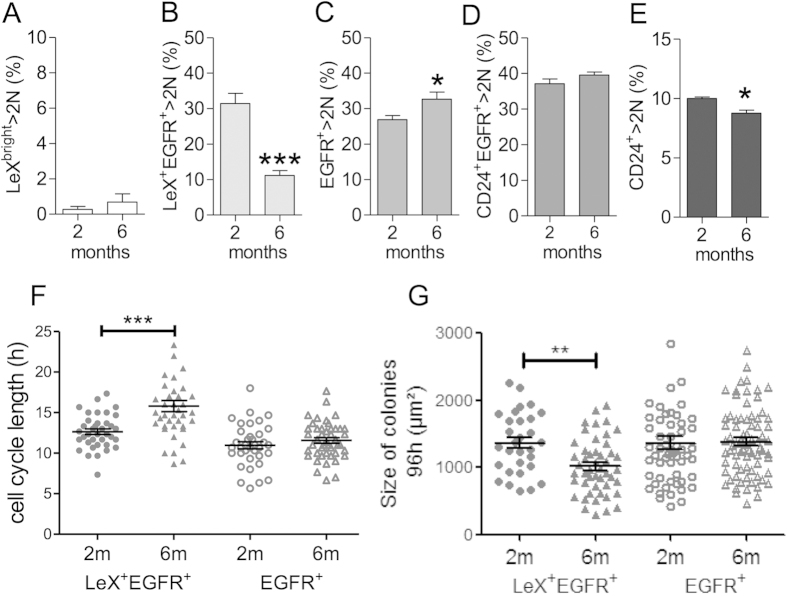
NSCs specifically exhibit proliferation defects at 6 months. (**A–E**) Proliferation status (S-G_2_/M %) of the SVZ populations of 2- and 6-month-old mice was determined by DNA content analysis using the vital DNA marker Hoechst 33342. (**F**) The length of the second division was determined by time-lapse videomicroscopy. (**G**) Colony size was measured 4 days after plating and provides an estimate of the amount of generated cells. The data are presented as the mean ± SEM and were obtained from at least 4 independent mice (**A–E**) and isolated cells from 2 independent groups of 3 to 5 mice (**F–G**) for each time point. *p < 0.05, **p < 0.01, ***p < 0.001.

To investigate further into the mechanism of neurogenesis disruption in young adults, we first compared the cell cycle status of the SVZ cell populations from 2- and 6-month-old mice by determining the S-G_2_/M fraction (DNA content > 2N) using the vital DNA marker Hoechst 33342^31^. In accordance with their quiescent status, LeX^bright^ cells had a low percentage of cells in the S-G_2_/M phase regardless of age (Fig. 2A). Strikingly, the percentage of cycling LeX^+^ EGFR^+^ cells was dramatically reduced by 6 months (Fig. 2B), whereas that of the EGFR^+^, CD24^+^ EGFR^+^, and CD24^+^ populations remained somehow stable or were moderately altered (Fig. 2C–E)^18^. Importantly, this decrease in aNSC proliferation was not associated with a permanent exit of the cell cycle as revealed by 14-day BrdU administration experiments (Fig. S2). Indeed, similar percentages of BrdU-positive cells were found among LeX^+^ EGFR^+^ cells at 2 and 6 months (Fig. S2).

We then used a colony-forming neurosphere assay^32^ to determine whether the decreased numbers of TACs at 6 months was related to a change in the clonogenic capacity of aNSCs and/or TACs. Interestingly, the quantification of primary neurospheres generated from LeX^+^ EGFR^+^ -sorted cells at 2 and 6 months showed no significant difference, confirming the absence of cell cycle exit of aNSCs at 6 months (Fig. S3).

We thus further investigated whether LeX^+^ EGFR^+^ cells had a defect in cell cycle progression using time-lapse video microscopy. Cell cycle lengths were determined during the second division after cell plating, taking advantage of culture conditions that maintain the cells in an undifferentiated state, i.e., in the presence of EGF and FGF2^18^,^29^,^33^. The cell cycle length of EGFR^+^ cells (TACs) was not altered between 2 and 6 months (Fig. 2F), leading to colonies with comparable sizes 4 days after plating (Fig. 2G). In contrast, the division of LeX^+^ EGFR^+^ cells (aNSCs) was lengthened by more than 3 hours in 6 month-old mice compared with 2-month-old mice (Fig. 2F; 12.6 ± 0.3 vs. 15.8 ± 0.7 hours, respectively), leading to significantly smaller colonies (Fig. 2G).

### Specific G_1_ lengthening in activated NSCs at 6 months

**Figure 3:**
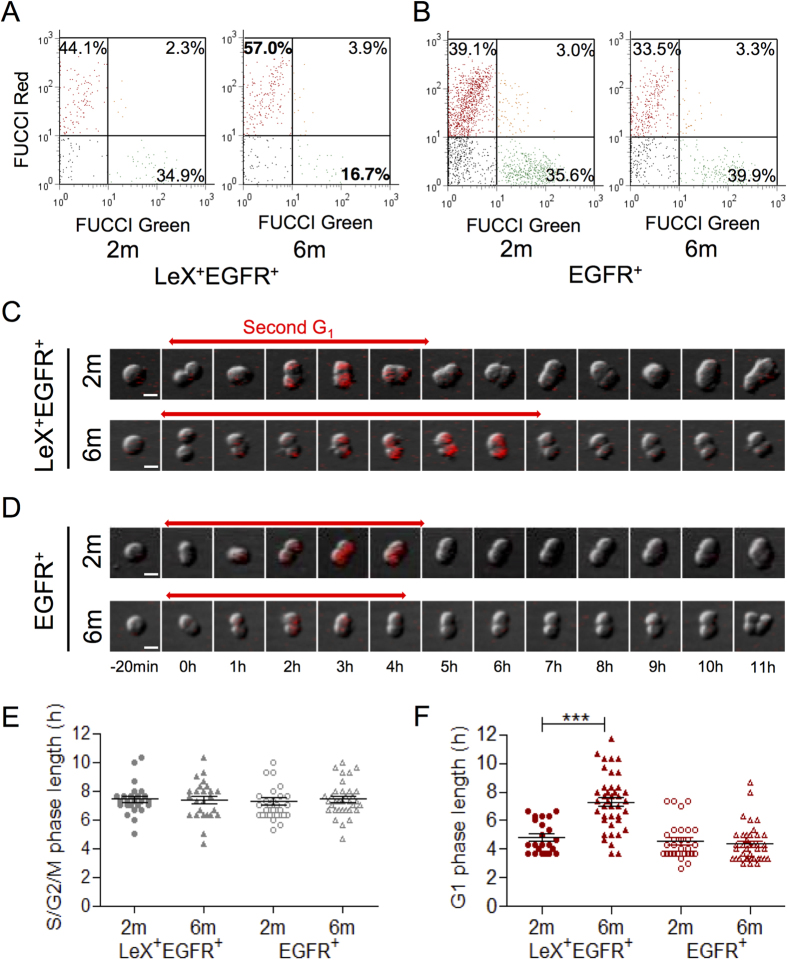
G_1_ lengthening in activated NSCs at 6 months. FUCCI mice (red and green fluorescent) were used to evidence an increase of the G_1_ proportion (FUCCI-Red cells) associated with a decrease of the S-G_2_/M proportion (FUCCI-green cells) specifically in (**A**) LeX^+^ EGFR^+^ cells, but not in (**B**) EGFR^+^ cells in 6-month-old mice. Videomicroscopy of (**C**) LeX^+^ EGFR^+^ and (**D**) EGFR^+^ cells sorted from FUCCI-Red mice allowing the tracking of the G_1_ phase with red fluorescence and the S-G_2_/M phase (colourless). (**E**) LeX^+^ EGFR^+^ and EGFR^+^ cells presented a similar S-G_2_/M phase length at 6 months, whereas (**F**) LeX^+^ EGFR^+^ cells had a longer G_1_ phase. The data are presented as the mean ± SEM and were obtained from analyses of cells isolated from at least 3 independent mice (**A,B**) and isolated cells from 2 independent groups of 3 to 5 mice (**C–F**) for each time point. *p < 0.05, ***p < 0.001. Scale bar for microscopy, 10 μm.

To explore the cell cycle of aNSCs and TACs in more detail, we used Fluorescence Ubiquitination Cell Cycle Indicator (FUCCI) mice^18^,^29^,^30^. FUCCI-Red cells allow for the visualization of the G_1_ phase through a G_1_ specific red-Cdt1 reporter, whereas FUCCI-Green cells exhibit green-fluorescent S-G_2_/M phases^30^. We found that a specific increase in the percentage of LeX^+^ EGFR^+^ cells in G_1_ (FUCCI-Red cells) was associated with a decrease of the S-G_2_/M fraction (FUCCI-Green cells) in 6-month-old mice compared with 2-month-old mice (Fig. 3A). As expected, these cell cycle alterations were not observed in EGFR^+^ cells (Fig. 3B).

Sorted cells from the FUCCI-Red mice were then plated under adherent conditions to visualize the cell-cycle phases at a single-cell level. A specific G_1_ lengthening was observed in LeX^+^ EGFR^+^ cells, rising from 4.8 ± 0.2 to 7.3 ± 0.3 hours at 2 and 6 months, respectively (Fig. 3C,F), whereas the S-G_2_/M phase was found unchanged (Fig. 3E). Again, no difference was observed between the EGFR^+^ cells from 2- and 6-month-old mice (Fig. 3D–F), confirming the lack of change in the proliferative capacities of TACs, even though the pool of these cells is dramatically reduced by 6 months.

The overall lengthening of the cell cycle observed in aNSCs at 6 months (Fig. 2F) nicely correlates with their increased percentage of FUCCI-red cells (Fig. 3A) and with the increased length of their G_1_ phase (Fig. 3F). Our results therefore hint at a causal factor that arises from cell cycle changes within the aNSC pool but not in their progeny, leading to a progressive neurogenesis decline. We previously reported proliferative defects coupled with G_1_ lengthening in aNSCs from 12-month-old mice^18^. To our knowledge, it is the first study to report such alterations as early as 6 months.

### Distinct molecular signature of 6 month-old aNSCs: modulation of the cell cycle machinery and the TGFβ signalling pathway

**Figure 4:**
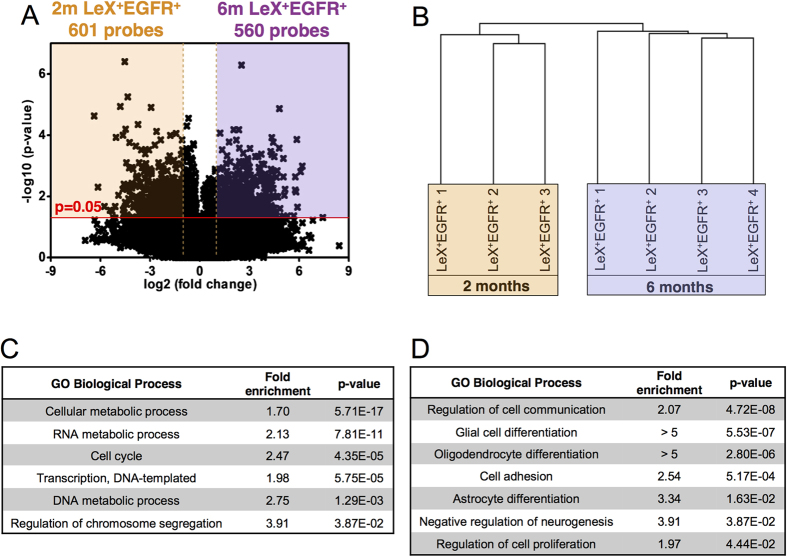
Microarray analysis reveals a distinct molecular signature of activated NSCs at 6 months. (**A**) Volcano plot of differentially expressed probesets in LeX^+^ EGFR^+^ cells from 2- (orange) and 6-month-old (purple) mice. (**B**) Hierarchical clustering of the expression data from all the biological replicates shows high reproducibility of the sortings and distinct transcriptional signature of LeX^+^ EGFR^+^ cells from 6-month-old mice. (**C,D**) Selected sets of significantly enriched GO categories using a statistical overrepresentation test for differentially expressed genes (p < 0.05) in (**C**) 2- and (**D**) 6-month-old LeX^+^EGFR^+^ cells.

To investigate the molecular mechanisms occurring in aNSCs that may cause the progressive decline in neurogenesis in the young adult, we performed a microarray analysis using whole-genome Affymetrix MOE430 2.0 arrays on LeX^+^ EGFR^+^ cells sorted from 2-month-old and 6-month-old mice. The comparative gene expression profile revealed an altered expression of 1,045 genes (Fig. 4A). The resulting set of LeX^+^ EGFR^+^ -enriched genes in 2-month-old mice included 562 genes (601 probe sets, Table S1), whereas 483 genes were upregulated at 6 months (560 probe sets, supplementary dataset 1). Hierarchical clustering of the expression data from all of the biological replicates resulted in the separation of the two cell populations (Fig. 4B), highlighting the distinct transcriptomic signature of 6-month-old LeX^+^ EGFR^+^ cells.

A Gene Ontology (GO) term analysis was then performed using a statistical overrepresentation test in PANTHER with Bonferroni correction^34^ to identify GO terms associated with each cellular state. As expected due to their highly proliferative status, the LeX^+^ EGFR^+^ cells from young mice were enriched in the GO categories of “cell cycle” and “regulation of transcription”^6^,^35^ (Fig. 4C; see supplementary dataset 2 for the complete list of GO terms and associated p-values). Moreover, the LeX^+^ EGFR^+^ cells from 6-month-old mice showed an overrepresentation of processes such as “negative regulation of neurogenesis” and “regulation of cell proliferation”, which was consistent with the modification of the neurogenic and proliferative properties of aNSCs described above (Fig. 4D; see supplementary dataset 2). In addition, several significantly enriched GO categories were linked to differentiation processes at 6 months (Fig. 4D).

**Figure 5:**
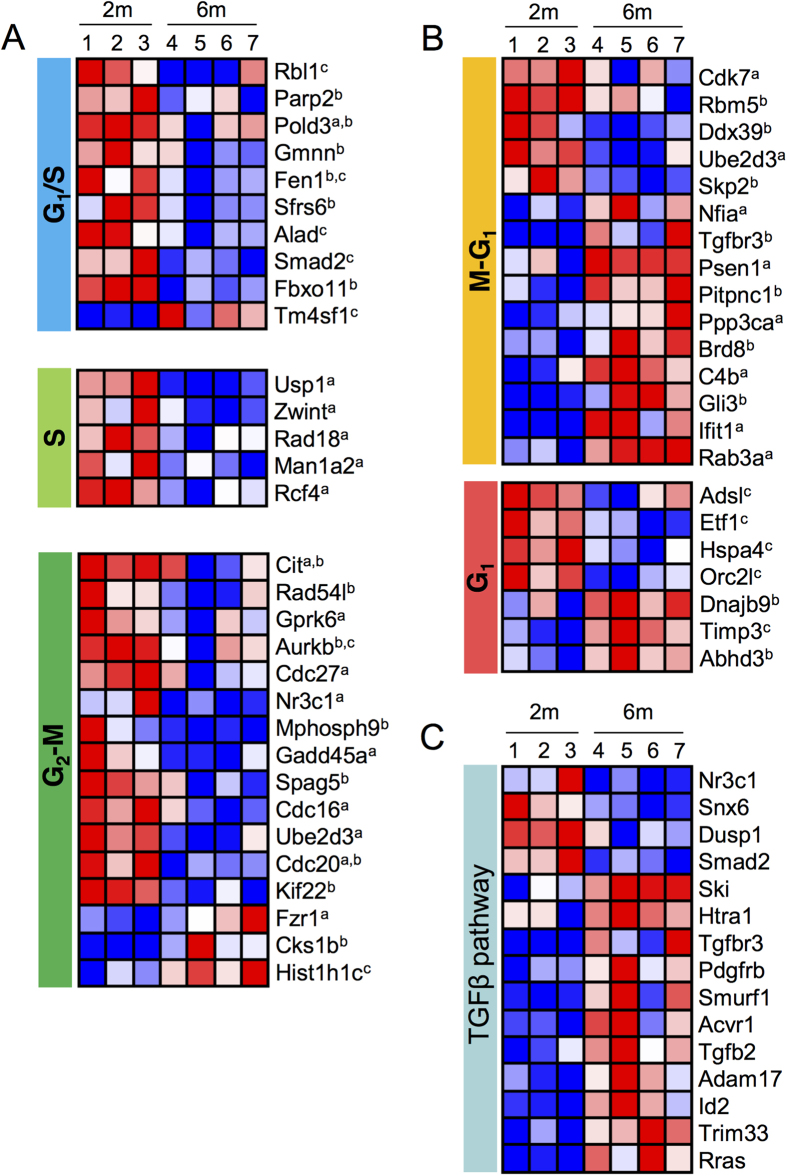
Genes implicated in the cell cycle regulation and the TGFβ pathway are modulated at 6 months in activated NSCs. Heat maps showing differences in transcript levels in LeX^+^EGFR^+^ cells from 2- and 6-month-old mice. Blue colour indicates low expression, and red indicates high expression (log2 scale). Replicate samples are shown for each group. (**A,B**) Cell cycle specific transcripts were classified by the cell cycle phase according to either ^a^Whitfield *et al.*^36^, ^b^Bar-Joseph *et al.*^37^ or ^c^Ishida *et al.*^38^. (**B**) Modulation of the TGFβ pathway.

We then investigated whether the cell cycle-related transcriptomic changes between 2- and 6-month-old aNSCs corroborated the cell cycle alterations observed *ex vivo* at 6 months. Among the differentially expressed genes associated with the cell cycle, 50 have literature references to specific phases, including the G_1_/S transition, the S phase, the G_2_-M phases or the M-G_1_ phases (Fig. 5A,B)^36^,^37^,^38^. Note that a majority of the genes was assigned to 2 consecutive phases, such as M-G_1_, as their transcripts were previously found to be persistently expressed over 2 distinct phases of the cell cycle^36^,^37^. Interestingly, we found that 67.6% of the cell cycle genes enriched in LeX^+^ EGFR^+^ cells from 2-month-old mice were linked to the S-G_2_/M phase (Fig. 5A) in accordance with their proliferative status, as shown above (Figs 2B and 3B). On the other hand, a large majority (81.3%) of the 6-month enriched cell cycle genes were associated with the G_1_ phase (Fig. 5A,B), which is also in concordance with the G_1_ lengthening observed at 6 months in the LeX^+^ EGFR^+^ cells (Fig. 3). Finally, the enrichment in transcripts linked to the G_1_/S transition in aNSCs from the 2-month-old mice further correlated with the altered cell-cycle dynamics of aNSCs at 6 months (Fig. 5A).

We have recently demonstrated an increase in TGFβ1 secretion in the aged (12–24 month-old) mouse SVZ that negatively impacts NSC proliferation, whereas a selective blockade of TGFβ signalling restores neurogenesis in aged mice^18^,^24^. TGFβ signalling in NSCs was thus shown as a key player in the regulation of neurogenesis during aging through inhibitory signals. We investigated whether this pathway was also modulated between cells isolated from 2- and 6-month-old mice. Consistent with the modulation of the TGFβ signalling pathway, transcripts of the genes in this pathway were upregulated in LeX^+^ EGFR^+^ cells isolated from 6-month-old mice (Fig. 5C). For example, TGFβR3 is overexpressed by aNSCs at 6 months in association with an endogenous overexpression of TGFβ2, suggesting a possible autocrine mechanism linked to the TGFβ signalling in aNSCs that could be involved in their lengthening of G_1_. HtrA1, which is a member of the HtrA serine protease family, has the ability to repress TGFβ signalling^39^,^40^ and has been shown to promote TGFβ1 degradation^41^. Interestingly, HtrA1 is upregulated at 6 months and could therefore counteract, to some extent, the role of TGFβ in age-related neurogenesis decline.

Our microarray analysis corroborates the cell cycle lengthening observed *ex vivo* and *in vitro* in aNSCs from young adult mice. Our analysis also suggests that the modulation of the TGFβ signalling pathway occurs earlier than previously anticipated, hinting at its potential role in an early neurogenesis decline.

## Discussion

Our study demonstrates that one of the first main events leading to a major decrease in the number of SVZ progenitor cells and, consequently, adult neurogenesis occurs between 2 and 6 months in mice and is related to a deregulation of the NSC cell cycle machinery. Although the pools of TACs and Im. Nbs are already reduced by 4 months, no change in their proliferative capacities was observed, hinting at a causal factor arising from the aNSC pool. Indeed, aNSCs specifically exhibit a proliferation deficit with the lengthening of their G_1_ phase, resulting in the decrease of progenitor cells at 6 months. Our gene expression profile analysis reveals their distinct transcriptomic signature compared with their 2-month-old counterparts and the modulation of the TGFβ signalling pathway. Together, our data highlight the importance of the NSC cell cycle regulation across the adult lifespan in mice and provide an attractive data resource for the development of regenerative therapies based on stimulating endogenous NSCs.

### Alteration of activated NSC proliferation is a main event leading to early neurogenesis decline

The recent emergence of FACS strategies has allowed the isolation and characterization of the different cell populations of the SVZ^4^,^5^,^6^,^8^,^25^,^26^. Using our published FACS method that allows for the characterization of 5 main neurogenic populations from the SVZ niche^5^,^29^, we showed that the pools of qNSCs and aNSCs were relatively stable until 12 months, in contrast to those of progenitor cells, which drastically decreased. Few studies have reported an alteration of NSCs within the first months, and they agree on the fact that the pool of NSCs is not altered between 2 and 6 months, either by FACS or pinwheel counting (present study^16^,^23^). Consistent with our results, and based on NSC apical GFAP-expressing process across the ventricle surface in a “pinwheel architecture”^42^, Shook *et al.* reported that the number of NSCs/mm^2^ is stable between 3 months and 6 months^23^. Similarly, reduced neurogenesis during aging in the adult hippocampus has been linked to an overall reduction of NSC proliferation rather than a loss of NSCs^21^. However, compelling evidence indicate that the pool of NSCs is reduced in elderly mice (24 month-old)^43^ suggesting that it is irreversibly depleted between middle-aged and elderly mice.

Few studies have explored the cell cycle of NSCs and their progenitors in the adult mouse brain *in vivo*^44^ or *in vitro*^18^,^33^. Our study shows that major intrinsic changes in the proliferation of aNSCs occur as early as 6 months within the SVZ. Indeed, combining acute FACS sorting and the use of FUCCI mice coupled with time-lapse video microscopy demonstrates for the first time that the cell cycle lengthening observed at 6 months is specific to aNSCs and not TACs and only impacts the G_1_ phase of the cell cycle. While the total cell cycle length of TACs is not altered a higher proportion of cells are in S-G2/M phase, probably to compensate the large decrease in aNSC proliferation (Fig. 2B,C). The aNSC G_1_ lengthening is progressive, as the cell cycle is lengthened by 3 hours between 2- and 6-month-old mice (present study) and by 6.5 hours between 2- and 12-month-old mice^18^. Moreover, despite evidence that associates the G_1_ lengthening in neural progenitors with a differentiation commitment during brain development^45^, in our study, the observed aNSC G_1_ lengthening was not associated with differentiation as only cells undergoing subsequent divisions were analysed, and thus, not cells exiting the cell cycle. Therefore, this G_1_ lengthening apparent by 6 months is specific to proliferative NSCs and results in a slowing down of the cell cycle, decreasing the number of generated cells. Our transcriptomic analysis of 2- and 6-month-old aNSCs further corroborated the cell cycle alterations observed at 6 months as a large majority of the genes enriched at 6 months and linked to the cell cycle were G_1_-specific genes, in contrast to those at 2 months. Our analysis also sheds light on a transcriptional signature of aNSCs at 6 months, at which significant changes are associated with cellular functions and pathways previously shown to play key roles in the regulation of neurogenesis within the SVZ.

### Pathways involved in early alteration of aNSCs

We recently reported an overproduction of TGFβ1 by the vascular niche in middle-aged mice, which leads to a neurogenesis decline that can be counteracted by selective anti-TGFβ blockage^24^. Using two different anti-TGFβ approaches to block TGFβ signalling, we demonstrated that TGFβ specifically lengthens the G_1_ phase in aNSCs but not in TACs from middle-aged mice^18^. The modulation of the expression of several actors of the TGFβ pathway observed here at 6 months in aNSCs suggests that this pathway could play a key role in the progressive loss of progenitor cells very early in the young adult. Unexpectedly, our transcriptomic analysis also suggests a possible autocrine regulation through the synthesis of TGFβ2 in aNSCs. Importantly, the maintenance of the aNSC pool over time and previous results on anti-TGFβ strategies suggest that aNSCs can be recruited to stimulate neurogenesis^16^,^18^,^21^. Our transcriptomic data could therefore help identify the signalling pathways that are altered with age in aNSCs, which may serve as future *in vivo* drug candidates in the context of aging and regenerative therapies based on stimulating endogenous NSCs. Due to the lack of data on early aging of SVZ neurogenesis, review articles have been forced to classify 2- and 6-month-old mice as “young adults”^46^. We suggest here that many dramatic changes happen during that time period at the stem cell level, making it necessary to study the early aging of neurogenic niches within the adult mammalian brain in more detail.

In summary, the neurogenesis decline observed during aging is a progressive process starting in the young adult mouse and is not caused by a loss of NSCs within the SVZ, as the pools of aNSCs and qNSCs remain stable with age. Instead, their progeny is dramatically reduced by the age of 4 months. Interestingly we report similar proliferative properties of TACs and Nbs at 2 and 6 months, whereas aNSCs exhibit a specific G_1_ lengthening at 6 months, a finding corroborated by our transcriptional study. Our study provides a solid data-resource for the identification of gene regulatory networks implicated in the regulation of adult neurogenesis.

## Additional Information

**How to cite this article**: Daynac, M. *et al.* Age-related neurogenesis decline in the subventricular zone is associated with specific cell cycle regulation changes in activated neural stem cells. *Sci. Rep.* **6**, 21505; doi: 10.1038/srep21505 (2016).

## Supplementary Material

Supplementary Figures S1-S3

Supplementary Table S1

Supplementary Table S2
