# Supplementary material for: Age-related neurogenesis decline in the subventricular zone is associated with specific cell cycle regulation changes in activated neural stem cells
Source: Sci Rep. 2016 Feb 19;6:21505. doi: 10.1038/srep21505 (PMC4759590; doi:10.1038/srep21505)

Supplementary information

Age-related neurogenesis decline in the subventricular zone is associated with specific cell cycle regulation changes in activated neural stem cells

Mathieu Daynac [1234*], Lise Morizur [1234*], Alexandra Chicheportiche[1234], Marc-André Mouthon[1234], François D. Boussin[1234]

Supplementary Figure Legends

Figure S1. Decrease in total SVZ cell number with aging is associated with a disorganization of the niche. (A) Evolution of the total number of cells in the SVZ during aging. (B-F) Quantification of the proportion of the main neurogenic populations relative to the total number of cells in the SVZ in mice aged 2 to 12 months. The data are represented as the mean±SEM and were obtained from at least 4 independent mice for each time point. * $p<0.05$, ** $p<0.01$, *** $p<0.001$

Figure S2. No permanent cell cycle exit at 6 months for activated NSCs and TACs. Frequency of BrdU-positive LeX^+EGFR^+ and $EGFR^+$ cells following a 14-day BrdU administration in 2- and 6-month-old mice (n=3, mean±SEM).

Figure S3. Neurosphere formation of activated NSCs and TACs is not altered at 6 months. The number of primary neurospheres was counted 7 days after plating LeX^+EGFR^+ and $EGFR^+$ sorted cells from 2- and 6-month-old mice in the presence of EGF and FGF-2 (n=4, mean±SEM).

Supplementary dataset legends (supplementary .xls files)

Table S1: Overrepresentation test and related GO categories of genes enriched in aNSCs from 2 month-old vs 6 months-old mice

Table S2: List of genes enriched in aNSCs from 2 month-old vs 6 months-old mice

Figure S1

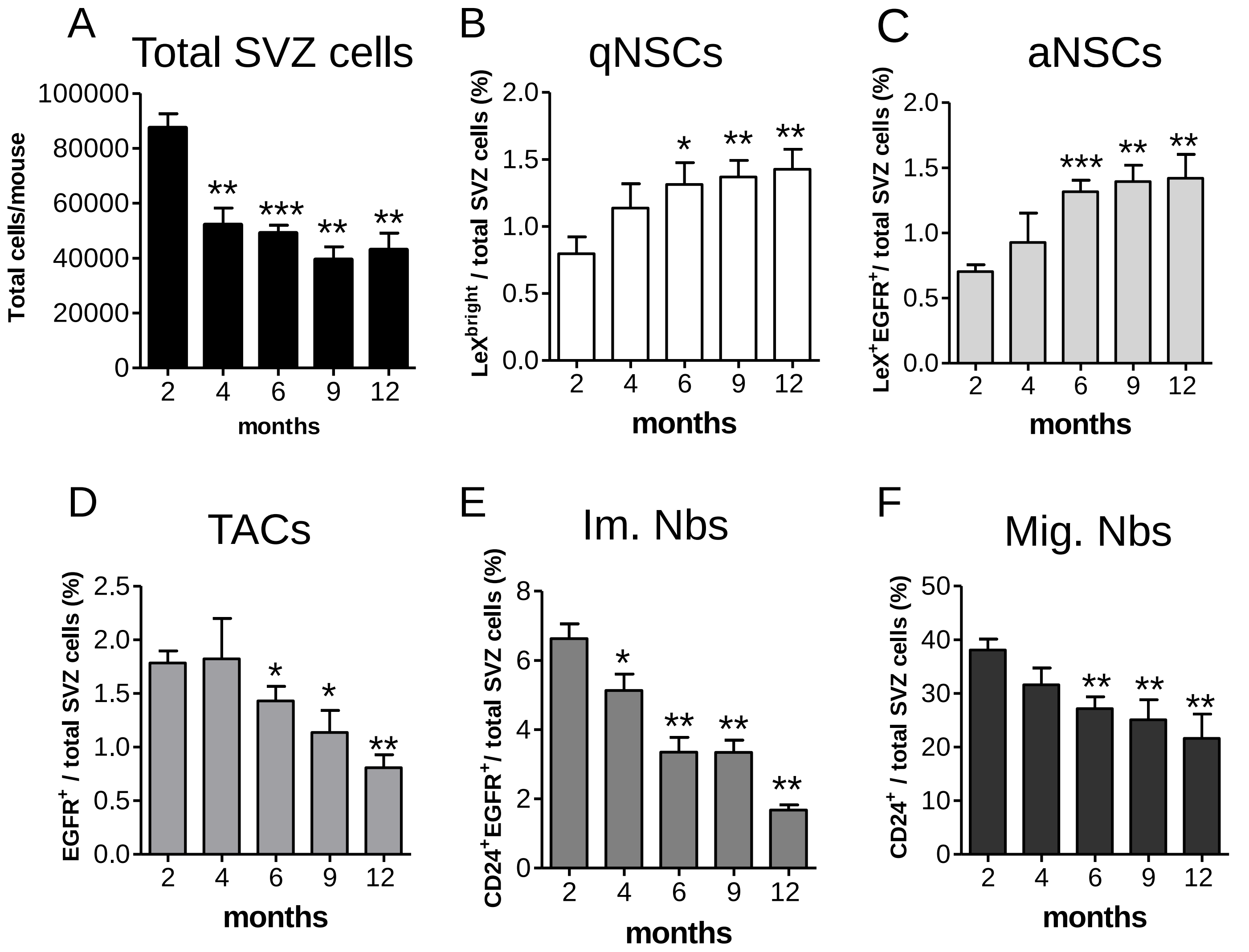
A
Total SVZ cells
Total cells/mouse
100000
80000
60000
40000
20000
0
**
***
**
**
2 4 6 9 12
months
B
qNSCs
LeX^bright / total SVZ cells (%)
2.0
1.5
1.0
0.5
0.0
*
**
**
2 4 6 9 12
months
C
aNSCs
LeX+EGFR+/ total SVZ cells (%)
2.0
1.5
1.0
0.5
0.0
***
**
**
2 4 6 9 12
months
D
TACs
EGFR+ / total SVZ cells (%)
2.5
2.0
1.5
1.0
0.5
0.0
*
*
**
2 4 6 9 12
months
E
Im. Nbs
CD24+EGFR+/ total SVZ cells (%)
8
6
4
2
0
*
**
**
**
2 4 6 9 12
months
F
Mig. Nbs
CD24+ / total SVZ cells (%)
50
40
30
20
10
0
**
**
**
2 4 6 9 12
months

Figure S2

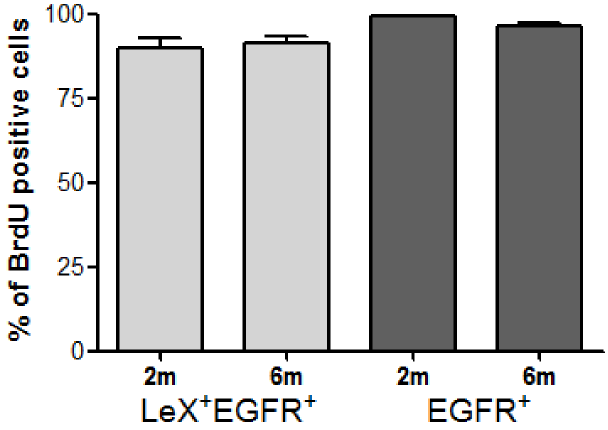

Figure S3

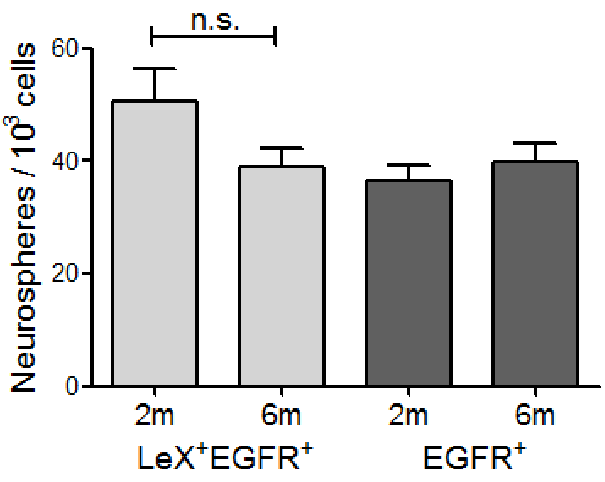

Supplement: Supplementary Figures S1-S3 [file srep21505-s1.pdf]
